# Preload Influence on the Dynamic Properties of a Polyurethane Elastomeric Foam

**DOI:** 10.3390/polym16131844

**Published:** 2024-06-28

**Authors:** Julen Cortazar-Noguerol, Fernando Cortés, Imanol Sarría, María Jesús Elejabarrieta

**Affiliations:** Department of Mechanics, Design and Industrial Management, University of Deusto, Avda. de las Universidades 24, 48007 Bilbao, Spain; fernando.cortes@deusto.es (F.C.); isarria@deusto.es (I.S.); maria.elejabarrieta@deusto.es (M.J.E.)

**Keywords:** preload, frequency, fractional model, dynamic properties, linear viscoelasticity, polymeric foam

## Abstract

Polymeric foams are widely used in engineering applications for vibration attenuation. The foams usually work preloaded and it is known that the dynamic properties and attenuation ability of these polymers depend on the preload. In this paper, experimental characterization of a polyurethane elastomeric foam is performed in a frequency range between 1 and 60 Hz, a temperature range between −60 and 30 °C and a preload range between 2 and 12 N, using a Dynamic Mechanical Analyzer. When going from the minimum to the maximum preload, results show the linear viscoelastic range increases 57%. In the frequency sweeps, the storage modulus increases 58% on average, while the loss factor remains unaffected by preload. Moreover, the glassy transition temperature of the material decreases for greater preloads. From the curve-fitting of a four-parameter fractional derivative model using the experimental data, a seven-parameter mathematical model is developed, reducing the number of parameters needed to describe the influence of frequency and preload on the dynamic properties of the material. Hence, it has been established that the relaxation time, relaxed modulus and unrelaxed modulus depend on the exponential of the squared prestress. In contrast, the fractional parameter does not depend on preload for the range under study.

## 1. Introduction

The problem of reducing vibrations for engineering applications can be tackled by means of different methods [1]. Among the passive methods [2], the addition of materials, as light as possible, has been proved to be a good way of absorbing energy and damping these vibrations. Polymeric foams offer, besides many other practical applications, a good example of those materials that, added to the original structure, can reduce the transmitted vibration [3,4,5,6,7]. When polymeric foams are used to reduce vibrations, preload is a crucial factor to be considered, since it may have a great influence in the dynamical properties of the material.

Current applications of polymeric foams include vibration damping in construction [8] or transport to improve comfort and durability. For instance, in the field of structural engineering, Ngamkhanong and Kaewunruen [9] proved the importance of the addition of an under sleeper pad (typically a polymeric foam) to the railway concrete sleepers. Regarding construction, Gatto et al. [10] performed resonant column tests to analyze the effects of polyurethane foams in seismic wave propagation.

This article focuses on a specific kind of high-performance polymeric foam: a microcellular polyurethane elastomer. As this material is viscoelastic, its dynamical behavior is determined by its complex modulus, which depends on several parameters, such as temperature, frequency, or, most important for the aim of the present work, the preload applied on it. Amongst the effects of these properties on the complex modulus, that of the applied preload is generally the least studied of them all.

The dependence of the mechanical properties of these materials on frequency is well known and there is also known work on the influence of preload on the mechanical behavior of elastomers. Hassan et al. [11] measured the effect of preload, among other properties, on the stiffness of elastomers used in automotive seats. Marvalova et al. [12] proposed an experimental procedure to study the influence of a multiaxial static preload on the dynamical properties of elastomers. Lectez et al. [13] showed that linearized models are sufficient to study this influence of the preload under small vibration amplitudes of the oscillations. Lion et al. [14] found that higher pre-deformations lead to a decrease in the elastomeric material’s stiffness and damping. Wollscheid et al. [15] studied the behavior of filler-reinforced rubber depending on the preload. Filho et al. [16] presented an approach to characterize simple elastomeric viscoelastic materials, including the frequency, temperature and preload effects, employing the Mooney–Rivling hyper-elastic theory to model the latter. El Maanaoui [17] developed a lifetime prediction for elastomer components with variable preload and temperature dependence. As for, specifically, polyurethane elastomers, Zhao et al. [18] studied mechanical properties such as the stress and the absorbing energy as a function of the strain in samples of these type of materials.

In terms of mathematical modelling of these properties, analytical models based on groups of springs and dashpots, such as Maxwell, Zener or Kelvin–Voigt differential models [19], are a first approximation. These models, however, tend to overestimate the slope of the loss modulus as a function of the frequency in the region of the glass transition [20]. Also, convolution integral-type models [21,22,23] use a relaxation function based on Boltzmann’s superposition principle. Adams et al. [24] used Prony series to analyze the dynamical properties of polymeric material. Torii et al. [25] loaded a polyurethane foam with different amplitudes and frequencies and modelled its dynamical properties using a non-parametric identification technique in the time domain. Fractional derivative models that combine standard dashpots, fractional dashpots and springs account for the behavior of viscoelastic materials [26,27]. A four-parameter fractional derivative model [28] has been proved to be very useful in determining the dynamical behavior of viscoelastic materials in the frequency domain. Meng et al. [29] investigated variable-order fractional viscoelastic models under various loading conditions. In this work, the four-parameter model is used.

Motivated by the importance of the effect of the preload in the dynamical properties of this particular polymeric foam, the aim of this paper is to study how the linear viscoelastic range and the complex modulus of the material are affected by preload and to propose a mathematical model than can describe the behavior of the dynamic properties of the material with both frequency and preload. 

The structure of the article is as follows: first, a theoretical review on the four-parameter fractional derivative model for viscoelastic materials is developed. Next, the experimental procedures are described, and the experimental results are shown. Then, a four-parameter fractional derivative model is fitted to each of the frequency sweeps. Finally, the dependence of each of the four parameters on the preload is described and accounted for, resulting in a seven-parameter analytical model for frequency and preload.

## 2. The Four-Parameter Fractional Derivative Model

In this section, a theoretical review of the four-parameter fractional derivative model for the dynamic mechanical properties of viscoelastic materials is presented. The four-parameter fractional derivative model describe the dynamic behavior of viscoelastic materials in the frequency domain with just four parameters. Moreover, the parameters hold a physical meaning, allowing for a significant analysis of the prestress effect on each of the parameters.

The dynamic behavior in the frequency domain of a viscoelastic material is described by the complex modulus [28], which represents the linear relationship between the applied stress and the resulting strain, and can be written as

(1) $$E^{*}(\omega )=\frac{\sigma^{*}(\omega )}{\varepsilon^{*}(\omega )},$$

where $\sigma^{*}$ and $\varepsilon^{*}$ are the Fourier transforms of the time-dependent stress and strain functions respectively, and ω is the angular frequency. The asterisk in each of the magnitudes represents their complex character. The linear behavior between stress and strain represented in Equation (1) is limited to a range of application. This range is known as the linear viscoelastic region (LVR). The LVR of a material is defined as the region where the strain shows a linear dependence on the stress applied [30].

It can be useful to decompose the complex modulus into its real and imaginary parts as

(2) $$E^{*}(\omega )=E^{\prime }(\omega )+iE^{\prime \prime }(\omega )=E^{\prime }(\omega )\left[1+i\eta (\omega )\right],$$

where $E^{\prime }$ is the storage modulus and represents the energy stored as an elastic deformation, $E^{\prime \prime }$ is the loss modulus and represents the energy loss on each of the oscillations due to viscous dissipation, $\eta =E^{\prime \prime }/E^{\prime }$ is the loss factor, the ratio between the loss and the storage moduli and $i^{2}=-1$ is the imaginary unit.

**Figure 1:**
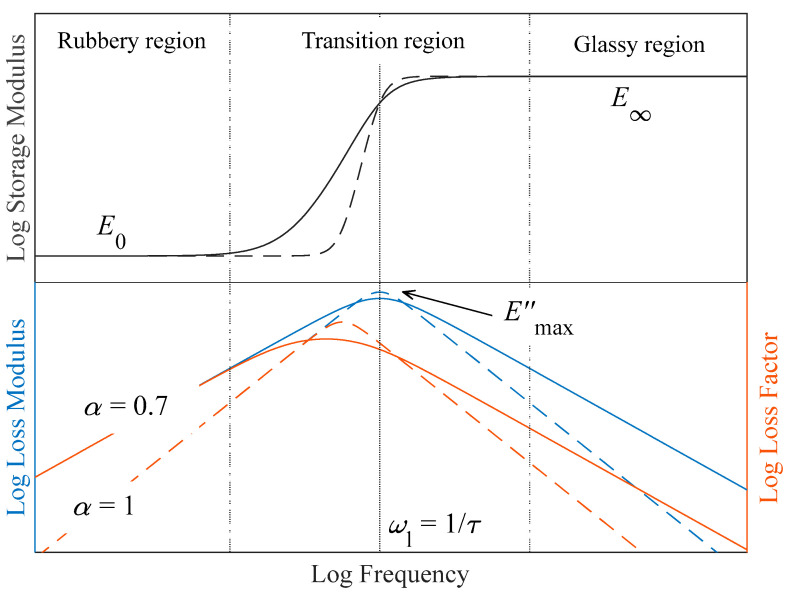
Complex modulus behavior with frequency in logarithmic scale. The Zener model is represented in dashed lines and a four-parameter fractional derivative model with *α* = 0.7 is presented in solid lines. Dashed-dotted vertical lines represent the limit between regions and the solid vertical line represents the glassy transition of the material.

Several mathematical models have been developed to describe the behavior of the complex modulus as a function of the frequency. The simplest models consist of ideally elastic springs and viscous dashpots, such as Kelvin–Voigt, Zener or Maxwell models. These models can be described by an arbitrarily large sum of time derivatives of increasing order [28]. Although this kind of model has been used for a long time, the qualitative behavior is far from the real behavior of materials. More specifically, the mentioned classical models tend to overestimate the slope of the loss modulus and the loss factor, as well as the slope of the storage modulus in the transition region (see Figure 1). This issue can be overcome by replacing the integer order derivatives with fractional ones. 

The *α*th order fractional derivative of a function ε(t) is defined with the gamma function Γ as [29]

(3) $$D^{\alpha }\left[\varepsilon (t)\right]=\frac{d^{\alpha }}{dt^{\alpha }}\varepsilon (t)=\frac{1}{\Gamma (1-\alpha )}\frac{d}{dt}\int_{0}^{t}\frac{\varepsilon (\tau )}{{\left(t-\tau \right)}^{\alpha }}d\tau ,$$

where τ is an integral variable. The fractional derivative equation describing the four-parameter fractional derivative model in the time domain is [28]

(4) $$\sigma (t)+bD^{\alpha }\left[\sigma (t)\right]=a_{0}\varepsilon (t)+a_{1}D^{\alpha }\left[\varepsilon (t)\right],$$

where $a_{0}$, $a_{1}$, b and α are the four parameters of the model. Performing a Fourier transform, Equation (4) can be translated to the frequency domain, resulting in

(5) $$\left[1+b{\left(i\omega \right)}^{\alpha }\right]\sigma^{*}(\omega )=\left[a_{0}+a_{1}{\left(i\omega \right)}^{\alpha }\right]\varepsilon^{*}(\omega ).$$

From Equation (5) and using the definition of complex modulus in Equation (1), the complex modulus of this model is given by

(6) $$E^{*}(\omega )=\frac{a_{0}+a_{1}{\left(i\omega \right)}^{\alpha }}{1+b{\left(i\omega \right)}^{\alpha }},$$

which is also known as the generalized Zener model. The classical Zenner model, also known as the Standard Linear Model in viscoelasticity, can be obtained by setting α=1 in Equation (6) as

(7) $$E^{*}(\omega )=\frac{a_{0}+a_{1}i\omega }{1+bi\omega }.$$

One of the benefits of the four-parameter fractional derivative model is that the four parameters have a physical meaning. Indeed, setting $a_{0}=E_{0}$, $a_{1}=E_{\infty }\tau^{\alpha }$ and $b=\tau^{\alpha }$, the complex modulus associated with the four-parameter fractional derivative model in Equation (6) is

(8) $$E^{*}(\omega )=\frac{E_{0}+E_{\infty }{\left(i\omega \tau \right)}^{\alpha }}{1+{\left(i\omega \tau \right)}^{\alpha }}=\frac{E_{0}\left[1+c{\left(i\omega \tau \right)}^{\alpha }\right]}{1+{\left(i\omega \tau \right)}^{\alpha }},$$

where $E_{0}$ is the relaxed modulus and represents the complex modulus value when the frequency tends to zero, $E_{\infty }$ is the unrelaxed modulus and represents the complex modulus value when the frequency tends to infinity, $c=E_{\infty }/E_{0}$ is the ratio between the unrelaxed and relaxed moduli, τ is the relaxation time of the material and α is the derivative fractional parameter (0<α<1), which is related to the slope of the storage modulus in the transition and the slope of the loss modulus and loss factor in the rubbery and glassy regions (see Figure 1). An α=0 value represents a purely elastic behavior, with no energy loss, while α=1 corresponds to the Zener model, represented in Equation (7). 

In Figure 1, a comparison between the Zenner model (Equation (7)) and the four-parameter fractional derivative model (Equation (8)) is shown. Three regions can be differentiated for the complex modulus. At low frequencies, in the rubbery region, the real part of the complex modulus (the storage modulus) remains almost constant, barely increasing. In this frequency range, the loss-factor and loss-modulus logarithm increase linearly with the logarithm of the frequency. Next, the transition region is reached. This region is characterized by an increase in the storage-modulus slope, followed by a change in its curvature sign. As for the loss modulus and the loss factor, in this region they reach their maximum value and begin to decrease. Both the maximum of the loss modulus and the change in the curvature sign of the storage modulus occur at a frequency $\omega_{l}=1/\tau$. The maximum of the loss factor is reached at a lower frequency, given by $\omega_{\eta }=\omega_{l}/c^{1/(2\alpha )}$ [28]. Finally, further increasing the frequency, the glassy region is reached. In this region, the storage modulus steadily tends to its unrelaxed value $E_{\infty }$. The loss-modulus and loss-factor logarithm decrease linearly with the frequency logarithm, and tend to zero.

While all three of the mentioned regions can be explored by manipulating frequency, the range depicted in Figure 1 typically spans over ten decades, rendering experimental access to these regions unfeasible. However, it is well-documented that an equivalence exists between frequency and temperature [31]. This equivalence dictates that a linear temperature increase (decrease) corresponds to an exponential decrease (increase) in frequency. All this considered, the experimental procedure to explore the different regions can be reduced to a temperature ramp at a constant frequency.

It is useful to define the different magnitudes presented in Figure 1 in terms of the four parameters of the fractional derivative model. The storage modulus is given by the real part of the complex modulus in Equation (8), namely,

(9) $$E^{\prime }(\omega )=\frac{E_{0}\left[1+(c+1)\cos\left(\frac{\pi \alpha }{2}\right){\left(\omega \tau \right)}^{\alpha }+c{\left(\omega \tau \right)}^{2\alpha }\right]}{1+2\cos\left(\frac{\pi \alpha }{2}\right){\left(\omega \tau \right)}^{\alpha }+{\left(\omega \tau \right)}^{2\alpha }}.$$

The loss modulus is the imaginary part of the complex modulus, which yields

(10) $$E^{\prime \prime }(\omega )=\frac{E_{0}(c-1)\sin\left(\frac{\pi \alpha }{2}\right){\left(\omega \tau \right)}^{\alpha }}{1+2\cos\left(\frac{\pi \alpha }{2}\right){\left(\omega \tau \right)}^{\alpha }+{\left(\omega \tau \right)}^{2\alpha }}.$$

In its maximum, the value of the loss modulus is [28]

(11) $$E_{\max}^{\prime \prime }=E^{\prime \prime }(\omega_{l})=\frac{E_{0}\left(\frac{c-1}{2}\right)\sin\left(\frac{\pi \alpha }{2}\right)}{1+\cos\left(\frac{\pi \alpha }{2}\right)}.$$

The loss factor can be obtained from the ratio between the loss and storage moduli, resulting in

(12) $$\eta (\omega )=\frac{(c-1)\sin\left(\frac{\pi \alpha }{2}\right){\left(\omega \tau \right)}^{\alpha }}{1+(c+1)\cos\left(\frac{\pi \alpha }{2}\right){\left(\omega \tau \right)}^{\alpha }+c{\left(\omega \tau \right)}^{2\alpha }}.$$

The aim of this work is to obtain a model able to describe the prestress $\sigma_{0}$ dependence of the complex modulus. To establish that dependence, the four-parameter fractional derivative model in Equation (8) is taken as a base, in which the constant four parameters are transformed into the following prestress-dependent functions

(13) $$\begin{array}{cccc} E_{0,\sigma }=E_{0,\sigma }(\sigma_{0}), & c_{\sigma }=c_{\sigma }(\sigma_{0}), & \tau_{\sigma }=\tau_{\sigma }(\sigma_{0}), & \alpha_{\sigma }=\alpha_{\sigma }(\sigma_{0}) \end{array}.$$

Therefore, the complex modulus associated with the prestress-dependent four-parameter fractional derivative model becomes

(14) $$E^{*}(\omega ,\sigma_{0})=\frac{E_{0,\sigma }\left[1+c_{\sigma }{\left(i\omega \tau_{\sigma }\right)}^{\alpha_{\sigma }}\right]}{1+{\left(i\omega \tau_{\sigma }\right)}^{\alpha_{\sigma }}}.$$

## 3. Materials and Methods

In this section, the material under study is described, as well as the equipment used and the experiments performed. Three different experiments are performed using a Dynamic Mechanical Analyzer (DMA) with a tension clamp. First, the linear behavior of the material must be tested. Then, a temperature ramp is performed to obtain the needed experimental data and finally, frequency sweeps are carried out in order to build and validate the proposed model.

### 3.1. Materials and Equipment

The material studied in this work is a microcellular polyurethane elastomer. To achieve this structure, certain blowing agents are added to an isocyanate and polyol mixture (also known as polyurethane precursor). These agents release gas when exposed to heat, creating bubbles within the material, which produces the microcellular structure. Depending on the amount and composition of the blowing agents and the heat and external pressure applied, different cell sizes, and therefore densities, can be obtained.

**Figure 2:**
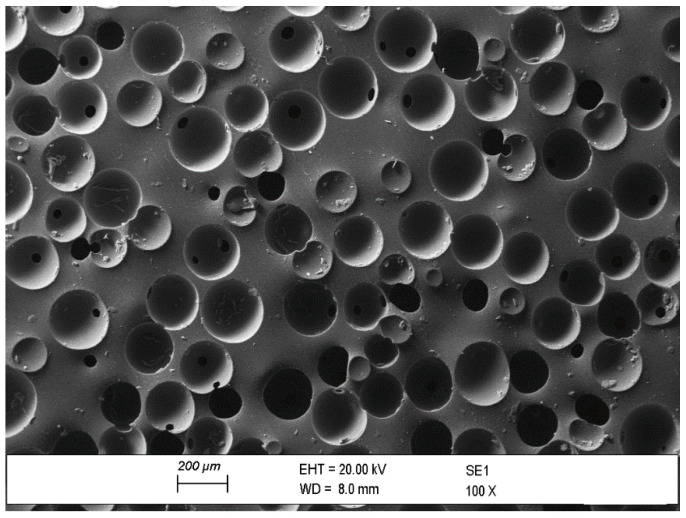
SEM image of the microcellular polyurethane elastomer under study. The image is taken with a ZEISS EVO50 microscope.

Figure 2 shows a Scanning Electron Microscope (SEM) image of the material under study using a ZEISS EVO50 microscope (ZEISS, Jena, Germany). The surface of the material is treated with a 5 nm thickness Au/Pd (80:20 weight ratio) layer. In this image, the microcellular structure of the material is revealed. The cell size of the structure varies between 100 µm and 200 µm.

**Figure 3:**
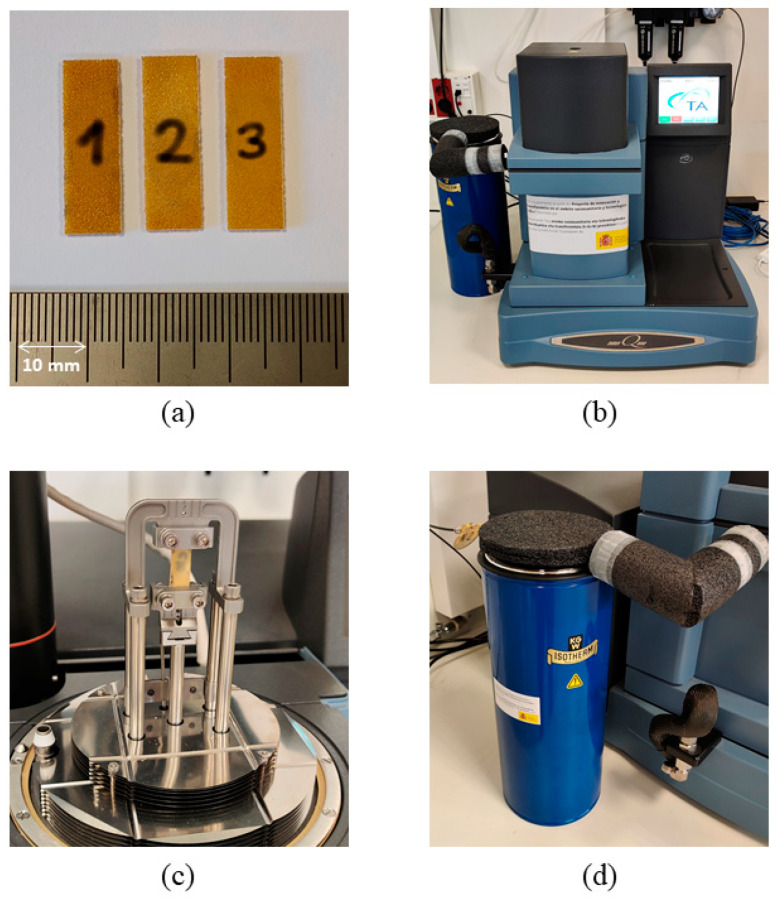
Photographs of (**a**) the test specimens, (**b**) the DMA, (**c**) the tension clamp and (**d**) the NPC accessory.

The test specimens received were already cut to meet the experimental needs of the DMA tension clamp used for the study. The geometrical characteristics of each of the 3 test specimens (Figure 3a) used can be checked in Table 1. To measure the width and the thickness of the samples, a digital Vernier caliper with a resolution of 0.01 mm has been used. Nevertheless, the length is not relevant, considering that the DMA machine measures the initial free length of the mounted specimen. Therefore, in Table 1 the nominal length is presented.

Every test performed in this work has been carried out using a DMA, specifically, the DMA Q800 model fabricated by T.A. Instruments (New Castle, DE, USA) (Figure 3b). This equipment can generate force-controlled oscillations in a frequency range from 0.01 Hz to 200 Hz with a force range from 0.001 N to 18 N. The minimum displacement the DMA can measure is 0.1 µm. For every test performed in this work, a tension clamp is used (Figure 3c). To carry out temperature ramps at sub-ambient temperatures, the Nitrogen Purge Cooler (NPC) accessory shown in Figure 3d has been used.

### 3.2. Experimental Methods

For the comprehensive characterization of the microcellular polyurethane elastomer, frequency sweeps and temperature ramps are essential. Due to the limitations of the DMA instrument, the experimental frequency range cannot be extended beyond 60 Hz for this material. Therefore, to obtain the material’s behavior in the transition and glassy regions, temperature sweeps are indispensable.

The parameters introduced into the DMA for tensile testing include preload force and force amplitude, as well as the ranges of frequencies or temperatures to be tested. Given the objective of this article, tests will be conducted with various preload forces. However, the force amplitude must remain constant across experiments and below the limit set by the LVR. Although the DMA experiments are configured with preload forces, the results of the temperature ramps and the frequency sweeps are presented in terms of prestress in the Experimental Results section.

**Figure 4:**
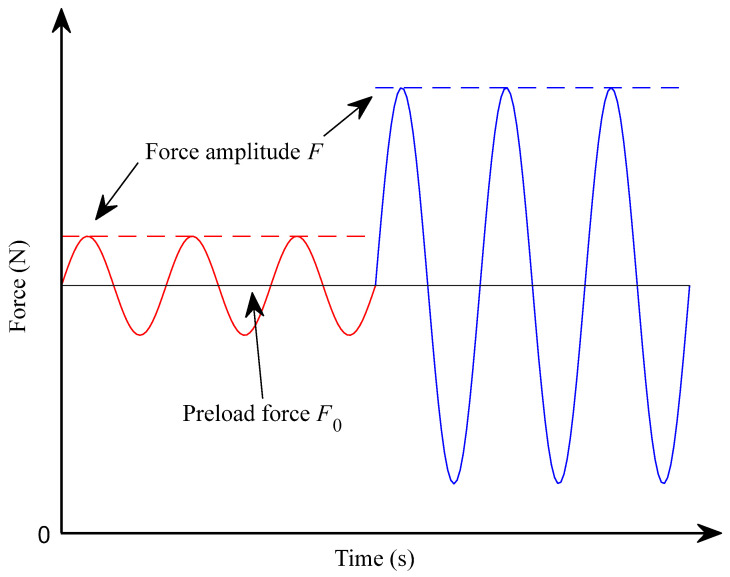
Depiction of the forces involved in a DMA tensile LVR test for a constant preload force (grey solid line) and the minimum (red dashed line) and maximum (blue dashed line) force amplitudes.

The LVR limit is defined as the point at which the complex modulus deviates significantly from its plateau value [21,32]. This limit is more restrictive when the material is less stiff, namely, at low frequencies or high temperatures [18]. Considering that the lowest frequency tested is 1 Hz and the highest temperature is 35 °C, the LVR test is performed under these conditions. Since the behavior of the material at different preload forces $F_{0}$ is as yet unknown, both the lowest (2 N) and highest (12 N) preload forces are tested. A depiction of the working procedure of the LVR test is shown in Figure 4. At the beginning of the test, the preload force $F_{0}$ (black horizontal line) and an initial force amplitude F (red line) are fixed. During the test, the force amplitude is gradually increased until it reaches its maximum (represented by the blue line), which must always be lower than the preload force applied to ensure that the sample is always under positive tension. For the lower preload force, the tested amplitudes range from 0.01 N to 0.9 N and for the highest preload force, the range is from 0.01 N to 3 N.

Once the force amplitude is fixed, the next step is to characterize the material. To this end, sub-ambient temperature ramps are performed at a constant frequency of 1 Hz for preload forces of 2 N, 8 N and 12 N. The temperature is first lowered to −60 °C and after a 10 min isothermal soak, is increased to 35 °C at a 3 °C/min rate. To decrease the temperature to the desired level, the NPC accessory is used (Figure 3d). From these tests, experimental values of $E_{0}$, $E_{\infty }$, $c=E_{\infty }/E_{0}$ and $E_{\max}^{\prime \prime }$ can be obtained for the tested preload forces. The fractional parameter α can also be calculated from Equation (11).

The frequency sweeps consist of a measurement of the complex modulus at different frequencies and for different preload forces with a fixed force-amplitude and temperature. The sweeps are performed under room temperature (23 °C) conditions, for preload forces of 2 N, 3.5 N and from 5 N to 12 N in 1 N steps. Three different sweeps for each preload force are preformed to ensure repeatability. The frequency range tested is from 1 Hz to 60 Hz in 5 Hz steps.

Although the frequency range is limited, this kind of test offers a fast and cost-efficient way of testing a wide range of preload forces. Moreover, the four-parameter fractional derivative model is meant to describe the complex modulus in terms of frequency, not temperature. Therefore, with the parameters obtained experimentally from the temperature ramps as initial values, a curve-fitting algorithm is used to fit the experimental data from the frequency sweeps, obtaining in this manner the parameter value across the whole preload force range tested. To do so, MATLAB’s Global Search Algorithm (GSA) is used [33] (MATLAB version 2023a).

## 4. Experimental Results

This section presents the obtained experimental results. First, the linearity test results, then the temperature ramps results, and, finally, the frequency sweeps results are presented.

### 4.1. Linear Viscoelastic Region

To test how the material behaves under different force amplitudes and preloads, a force-amplitude ramp is performed. Because the preload force can also change this behavior, the ramps will be performed using the lowest and the highest preload forces tested in this work. The goal of this experiment is to find the limit of the LVR of the material.

**Figure 5:**
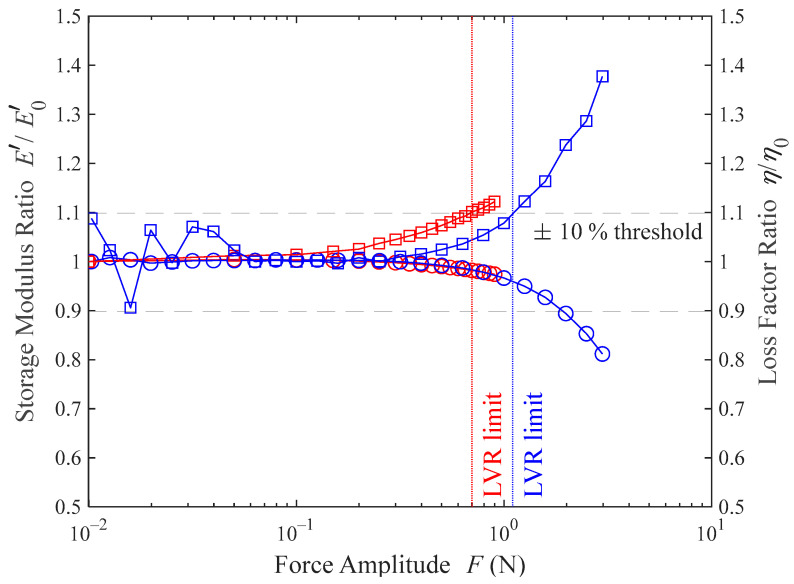
Storage modulus (○) and loss factor (□) variation, as a function of force amplitude, at 1 Hz frequency and 35 °C. Two different preload forces are applied: 2 N (red) and 12 N (blue).

In Figure 5 it is shown how the tested material behaves under different force amplitudes. For a better visualization, the normalized storage modulus (in circles) and loss factor (in squares) are plotted. This normalization is defined as the value of the observed magnitude divided by its value in the plateau region of the material. The dashed lines represent a 10% variation over the plateau value of the measured magnitude, and the vertical red and blue lines represent the force amplitude limit under a 2 N preload force and a 12 N preload force, respectively.

Although the usual criterion to define the LVR is based on the variation in the storage modulus, the loss factor variation happens to be more sensitive to amplitude increases [32]. For this reason, the LVR limit criterion followed in this work is a 10% change in the loss factor. From the results shown in Figure 5, the LVR limit can be set at 0.7 N force amplitude for a 2 N preload force, and at 1.1 N force amplitude for a 12 N preload force. These results prove that the LVR increases with the preload force and that the loss factor variation restricts this range more than the storage modulus variation, for which the limit would be set at 1.9 N force amplitude.

As a conclusion, for both the frequency sweeps and the temperature ramps, the force amplitude is set to 0.7 N. This choice is not arbitrary. Although a smaller force amplitude could be selected for the frequency sweeps, this is not the case for the temperature ramps. Cooling the material to its glassy region causes it to stiffen. Therefore, with the force amplitude remaining constant, the measured displacement amplitudes will decrease as rigidity increases. Since the DMA has a lower limit on the displacements it can measure (0.1 µm), maximizing the force amplitude enhances the precision with which the complex modulus is measured at low temperatures.

### 4.2. Temperature Ramps

At room temperature, the microcellular polyurethane elastomer under study is in its rubbery region. To completely explore the three viscoelastic regions described in Figure 1, a temperature ramp can be performed.

**Figure 6:**
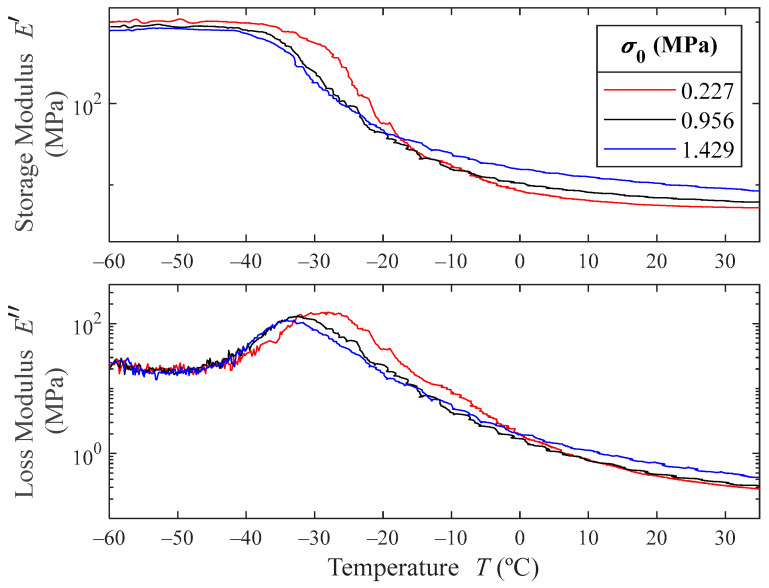
Experimental results for the storage and loss moduli at three different prestresses and 1 Hz as a function of temperature. For the sake of visualization, the scale is logarithmic.

Figure 6 shows the obtained storage- and loss-moduli results for the temperature ramps. The results show that at low temperatures, the storage modulus is bigger for lower prestresses, while at room temperature the behavior is the opposite. As expected, in the glassy region, the material greatly stiffens. The storage modulus in this region is between 100 and 200 times higher than in the rubbery region.

**Figure 7:**
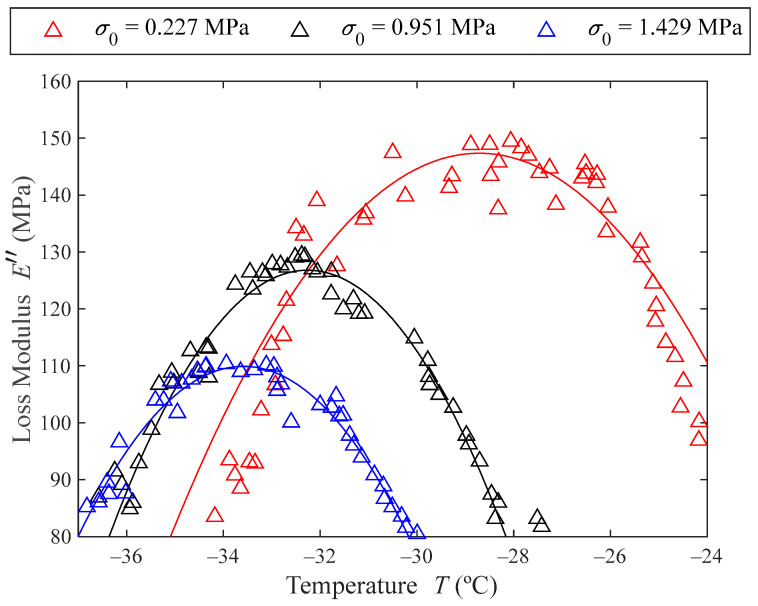
Loss modulus as a function of temperature for the three tested prestresses. The experimental data are represented with triangles (△) and the solid lines represent parabolic curve fits.

As can be pointed out in Figure 6 (and detailed in Figure 7), the temperature at which the loss modulus maximum occurs decreases with the prestress, meaning that the glassy transition temperature decreases with the prestress. The maximum value of the loss modulus also decreases with increasing prestresses, which means that the material decreases its damping capacity with greater prestresses.

From these experimental results, the experimental values of $E_{0}$, $E_{\infty }$ and $E_{\max}^{\prime \prime }$ can be obtained for the tested prestresses. $E_{0}$ is the minimum value of the storage modulus obtained from the temperature ramps, namely, in the rubbery region. Because at low temperatures the measurements are noisy, $E_{\infty }$ is obtained from the mean value of the storage modulus in the glassy region. From visual inspection of Figure 6, the limit of the glassy region for each prestress curve is set to −45 °C, and the maximum value of the storage modulus is set as $E_{\infty }=\mathrm{mean}\left[E_{\exp}^{\prime }(T)\right],\;\;\forall T\in [-60,-45]\;{}^{\circ}C$.

Once $E_{0}$ and $E_{\infty }$ are determined, $c=E_{\infty }/E_{0}$ can also be computed. Using Equation (11) an estimation for the value of α can be made. To do so, the maximum values of the loss factor for each of the tested prestresses must be found. Figure 7 shows the peaks of the loss modulus as a function of temperature. The triangles represent the experimental data points. Given the experimental data are slightly noisy, the peak of each prestress curve has been modelled by parabolas (represented with solid lines in Figure 7). The maximums of these parabolas give the maximum loss modulus $E_{\max}^{\prime \prime }$, also shown in Table 2.

Finally, solving Equation (11) for α with the values of $E_{0}$, c and $E_{\max}^{\prime \prime }$, the value for the fractional derivative exponential parameter is obtained and shown in Table 2.

The parameter values obtained in Table 2 give a quantitative description of the microcellular polyurethane foam in the three viscoelastic regions described in Figure 1. Nevertheless, we lack an experimental method that allows the calculation of τ for each prestress. However, it is possible to curve-fit the four-parameter fractional derivative model to the experimental data from frequency sweeps in order to obtain an estimation of this parameter.

### 4.3. Frequency Sweeps

**Figure 8:**
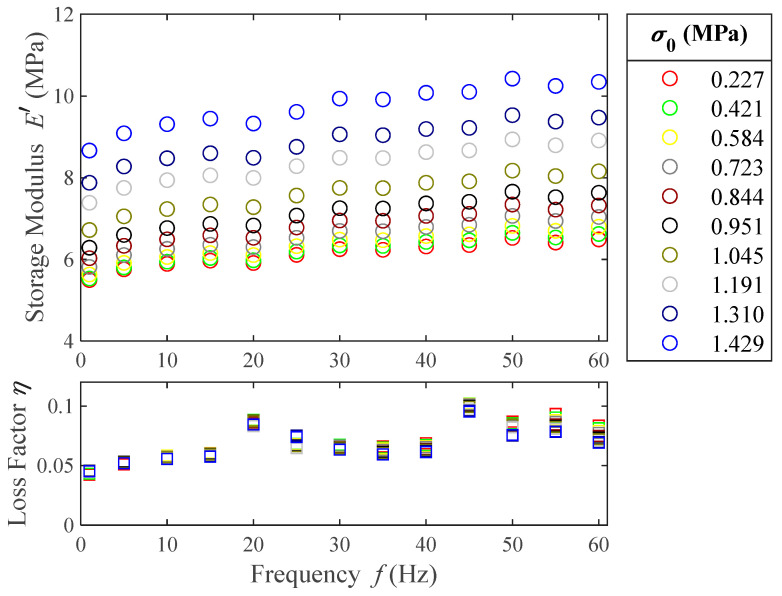
Experimental results of the storage modulus (○) and loss factor (□) for frequencies from 1 to 60 Hz and different prestresses. The color of each prestress is shared between storage modulus (○) and loss factor (□) markers.

The model that we aim for is frequency-dependent. Therefore, to perform the parametric fit of the four-parameter fractional derivative models, frequency sweeps are needed. Moreover, this parametric fit is the only method we can follow to estimate τ and these tests are cost-efficient and less time-consuming than temperature ramps. Hence, a greater set of prestresses can be tested. The frequency sweep results are shown in Figure 8.

From this figure it can be pointed out that the material shows stiffening when a higher prestress is applied. On average, the increase in storage modulus between the lowest and highest prestresses tested is 58%. However, the loss factor does not seem to significantly vary with the prestress, especially at low frequencies. Both the storage modulus and the loss factor of the material increase with the frequency, which shows that the polymer is in its rubbery viscoelastic region at room temperature, as was already established in the temperature ramps. The increase in storage modulus due to the frequency increase is 18% for the lowest prestress tested and 20% for the highest. The average increase in loss factor due to the frequency increase is 75%. From these results, it can be concluded that the storage modulus is mostly affected by prestress.

## 5. Prestress-Dependent Model

To build the prestress-dependent model, the experimentally obtained parameter values are used as a first parametric function approximation (Table 2). Among these functions $\tau_{\sigma }$ is the only one for which no data are available from the temperature ramps results, and it must be obtained directly from the curve-fitting of the frequency sweeps results. On the other hand, for the prestress functions $E_{0,\sigma }$, $c_{\sigma }$ and $\alpha_{\sigma }$, three experimental values are obtained from the temperature ramps, listed in Table 2. Nevertheless, taking into account the fact that ten prestress curves are available in the frequency sweeps (Figure 8), covering a more detailed range of prestresses than the temperature ramps, all the parameters of the prestress-dependent functions of Equation (13) will be obtained by a curve-fitting method, as presented in the next section. An important advantage of having knowledge of the results obtained in Table 2 is that they serve as initial values for the curve-fitting procedure, which is a key point in this type of methods.

### 5.1. Curve-Fitting Process

To approach the curve-fitting process, the MATLAB GSA functionality is used [33]. This algorithm allows for a simultaneous curve-fitting of the experimental data from the ten different prestresses tested, optimizing the value of forty parameters (four per prestress level tested).

The objective function to be minimized is defined as

(15) $$g(X)=\frac{1}{n}\overset{n}{\underset{i=1}{\sum }}\left[\frac{1}{m}\overset{m}{\underset{j=1}{\sum }}\left(\frac{\left|E_{i,j}^{\prime }-E^{\prime }(x_{i},\omega_{j})\right|}{\max(E_{i}^{\prime })}+\frac{\left|\eta_{i,j}-\eta (x_{i},\omega_{j})\right|}{\max(\eta_{i})}\right)\right],$$

where n is the number of prestresses tested, m is the number of frequencies tested; $E_{i,j}^{\prime }$ and $\eta_{i,j}$ are the experimental storage modulus and loss factor at a prestress $\sigma_{0,i}$ and a frequency $\omega_{j}$, respectively; $\max(E_{i}^{\prime })$ and $\max(\eta_{i})$ are the maximum values of the experimental storage modulus and loss factor at a $\sigma_{0,i}$ prestress, respectively; and

(16) $$X=\left\{x_{0},x_{1},\ldots ,x_{i}\right\}$$

is the full parameter matrix, where $x_{i}=\{E_{0,\sigma }(\sigma_{0,i}),\;c_{\sigma }(\sigma_{0,i}),\;\tau_{\sigma }(\sigma_{0,i}),\;\alpha_{\sigma }(\sigma_{0,i})\}$ represents the set of parameters that fulfil the fractional derivative model in Equation (14) at a $\sigma_{0,i}$ prestress. Finally, $E^{\prime }(x_{i},\omega_{j})$ and $\eta (x_{i},\omega_{j})$ are the storage modulus and loss factor given by Equations (9) and (12), respectively, with a set of parameters $x_{i}$ and a frequency $\omega_{j}$.

The GSA uses the following input parameters: $X_{0}$ is the 10×4 matrix of initial values for each of the model parameters. For this input, the values estimated for the parameters from the temperature ramps have been used except for τ, for which there is no possible educated guess from these tests. Therefore, a value of $\tau =10^{-10}\;s$ is set. Additionally, upper and lower bounds can be set for each of the parameters. These bounds act as constraints in the optimization process. For this problem, a 20% tolerance is set for $E_{0}$, c and α. For τ, a two-logarithmic decade margin has been set as tolerance.

With the optimization complete, the minimum value of the objective function is

(17) $$g(X_{\mathrm{optim}})=0.120,$$

which can be interpreted as the mean value of the normalized deviation of each data point, meaning, on average, the curve fitting deviates 12.0% from the experimental data. Given this average deviation from the experimental data, the curve fitting process is considered to accurately describe the experimental data, accounting for the effects of both frequency and prestress with ten different four-parameter fractional derivative models, resulting in the 40 independent parameters shown in Table 3.

**Figure 9:**
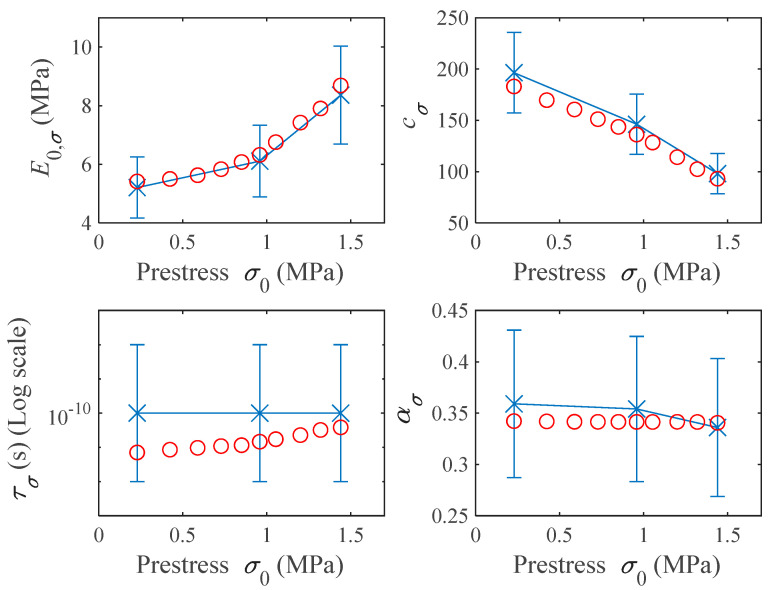
Representation of the four parameters of the fractional derivative model in terms of prestress. Blue crosses represent the initial values taken as inputs in the GSA. The blue bars represent the search bounds for each of the parameters. Red circles represent the curve-fitting results.

In Figure 9, both the initial value (including the search bounds as blue bars) and the curve-fitting result of each parameter are shown as a function of the prestress applied. It is worth noting that none of the 40 optimized parameters falls outside or near the boundary of the upper and lower bounds set in the GSA configuration. This is of great significance, as it indicates that the estimates derived from temperature ramps are consistent with the results obtained in frequency sweeps. From these results, it can be concluded that the $E_{0,\sigma }$ and $\tau_{\sigma }$ values increase with prestress while $c_{\sigma }$ decreases. For $\alpha_{\sigma }$ an almost constant value is obtained in the curve-fitting process.

Next, to generalize the obtained values to a continuous range of prestresses, and reduce the number of parameters needed to describe the experimental data, a mathematical model dependent on the frequency and prestress is developed.

### 5.2. Model Building

**Figure 10:**
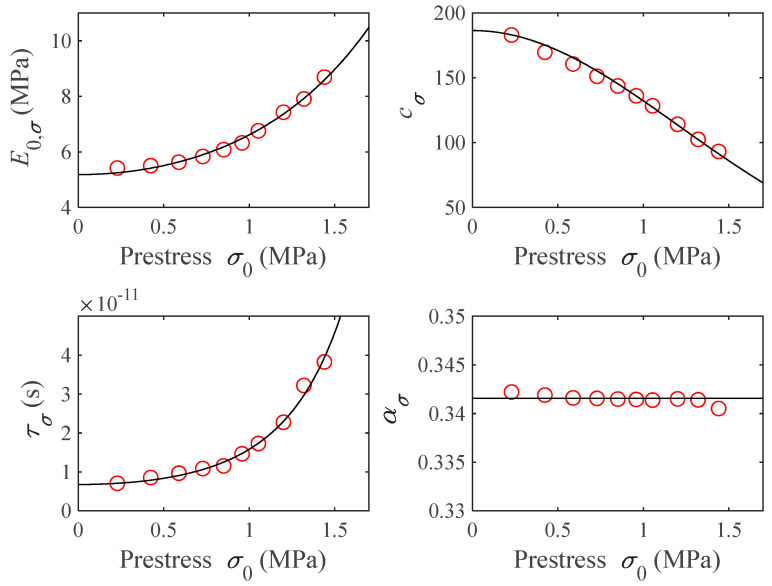
Fractional derivative-model parameters $E_{0,\sigma }$, $c_{\sigma }$, $\tau_{\sigma }$ and $\alpha_{\sigma }$ as a function of prestress $\sigma_{0}$. Red circles represent the optimization result and black lines represent the proposed models.

The resultant parameters of the curve-fitting process from the frequency curves (the results of Table 3) are represented in Figure 10 by red circles. By inspection of these data, a constant is proposed for $\alpha_{\sigma }$ and an exponential function of the prestress squared is proposed for $E_{0,\sigma }$, $c_{\sigma }$ and $\tau_{\sigma }$ as follows:

(18) $$\alpha_{\sigma }=\alpha_{0},$$

(19) $$E_{0,\sigma }=E_{0,0}\exp(\beta \sigma_{0}^{2})$$

(20) $$c_{\sigma }=c_{0}\exp(-\gamma \sigma_{0}^{2})$$

and

(21) $$\tau_{\sigma }=\tau_{0}\exp(\delta \sigma_{0}^{2}/\alpha_{0}).$$

With these functions, the values of the four parameters of the original fractional derivative model can be found again when no prestress is applied, namely, $E_{0,0}$, $c_{0}$, $\tau_{0}$ and $\alpha_{0}$, as well as the parameters β, γ and δ, which add the prestress dependence to the model. These seven parameters must be real and positive, and they can be obtained by fitting the Equations (18)–(21) to the respective values obtained in the curve-fitting process (Table 3). The results of these fitted seven parameters are presented in Table 4. It is worth mentioning that the three parameters accounting for the prestress effect β, γ and δ fall into the same magnitude order, which indicates a similar effect of the prestress in the three fitted functions $E_{0,\sigma }$, $c_{\sigma }$ and $\tau_{\sigma }$.

Figure 10 shows the obtained functions from Equations (18)–(21) when the parameter values shown in Table 4 are introduced in solid black lines. As it can be seen, these functions resemble, to a great extent, the values obtained in Table 3, shown as red circles in the same figure.

Finally, substituting the Equations (18)–(21) in Equation (14), the final prestress- and frequency-dependent model results in

(22) $$E^{*}(\omega ,\sigma_{0})=\frac{E_{0,0}\left\{1+c_{0}{\left(i\omega \tau_{0}\right)}^{\alpha_{0}}\exp\left[(\delta -\gamma )\sigma_{0}^{2}\right]\right\}\exp(\beta \sigma_{0}^{2})}{1+{\left(i\omega \tau_{0}\right)}^{\alpha_{0}}\exp(\delta \sigma_{0}^{2})},$$

which represents the model proposed in this work to address the effect of the frequency and prestress in the complex modulus of a viscoelastic material.

**Figure 11:**
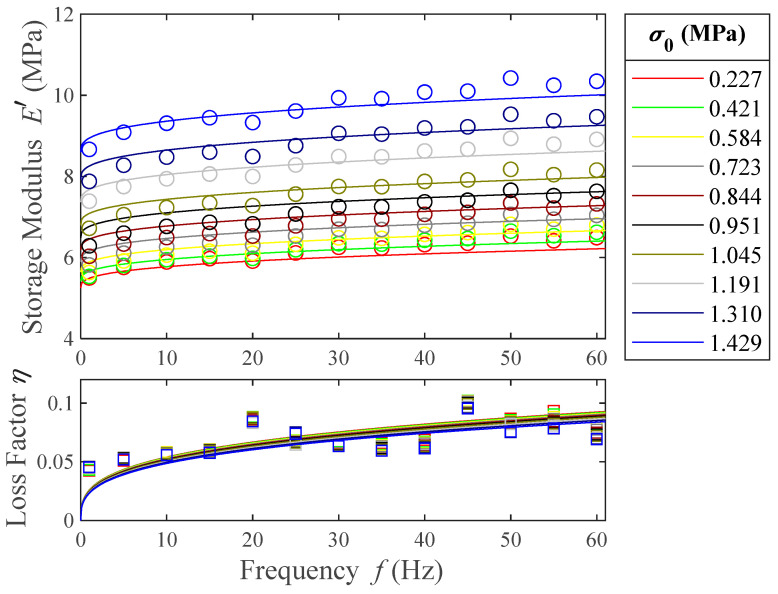
Experimental storage modulus (○) and loss factor (□) as a function of frequency. Solid lines represent the complex modulus given by the seven-parameter prestress-dependent model.

Figure 11 shows the comparison between the prestress-dependent model given by Equation (22) using the parameters in Table 4, and the experimental data, in order to verify the model validity. 

Finally, to validate the model, the objective function in Equation (15) is used. In this case, the analytical values of both the storage modulus and the loss factor are obtained from the proposed model in Equation (22). The mean normalized deviation obtained is a 12.5% with respect to the experimental data. Comparing this value to the one obtained in Equation (17) (12.0%), it can be concluded that the developed model describes the experimental data to the same extent as the independent four-parameter fractional derivative models fitted in Section 5.1, reducing the number of independent parameters from 40 to only 7.

## 6. Conclusions

In this study we have explored the dependence of the complex modulus of a viscoelastic material with both frequency and prestress levels applied. This knowledge is fundamental for the correct application in industry of vibration-attenuating materials that are subjected to preload. This investigation has yielded a mathematical model and several significant conclusions. The LVR increases with prestress. The loss factor variation defines the LVR. In addition, it has been proven that the storage modulus is bigger for lower preloads in the glassy region, while in the rubbery region the opposite is true. The glassy transition temperature decreases with the preload. Moreover, the frequency sweeps at different prestress levels show an increase of 58% in the storage modulus, while the loss factor remains constant in the prestress range tested.

On the other hand, the mathematical modelling of the experimental results allowed for the building of a seven-parameter fractional derivative model that describes the effect of frequency and prestress, to a great extent. The proposed model has been built by including the dependence on the prestress of the four-parameter fractional derivative model, generating a model that reduced the parameter count from forty to only seven. It has been established that, for the prestress range tested, the relaxed and non-relaxed modulus and the decay time depend exponentially on the square of the applied prestress, while the fractional derivative parameter can be considered constant. 

## Figures and Tables

**Table 1:** Geometrical properties of the microcellular polyurethane elastomer test specimens.

ID	Length (±1 mm)	Width (±0.01 mm)	Thickness (±0.01 mm)	Section Area (±0.1 mm^2^)
1	25	8.02	1.04	8.3
2	25	8.03	1.05	8.4
3	25	8.02	1.06	8.5

**Table 2:** Parameter estimation using the experimental data from the temperature ramps for different prestresses.

$F_{0}$ (N)	$\sigma_{0}$ (MPa)	$E_{0}$ (MPa)	$E_{\infty }$ (MPa)	$E_{\max}^{\prime \prime }$ (MPa)	c	α
2	0.227	5.21	1022.86	147.35	196.46	0.359
8	0.951	6.11	893.51	126.80	146.26	0.354
12	1.429	8.36	820.89	109.91	98.17	0.336

**Table 3:** Numerical values of each parameter after the curve-fitting process.

$\sigma_{0}$ (MPa)	$E_{0,\sigma }$ (MPa)	$c_{\sigma }$	$\tau_{\sigma }$ (ps)	$\alpha_{\sigma }$
0.227	5.417	183.1	7.07	0.3422
0.421	5.503	169.7	8.54	0.3419
0.584	5.630	160.6	9.66	0.3416
0.723	5.836	151.2	10.85	0.3416
0.844	6.080	143.7	11.56	0.3415
0.951	6.323	136.1	14.64	0.3414
1.045	6.758	128.4	17.29	0.3414
1.191	7.426	114.1	22.75	0.3415
1.310	7.905	102.5	32.21	0.3414
1.429	8.692	93.2	38.28	0.3405

**Table 4:** Prestress- and frequency-dependent model parameters for the tested material.

$E_{0,0}$ (MPa)	β (MPa^−2^)	$c_{0}$	γ (MPa^−2^)	$\tau_{0}$ (ps)	δ (MPa^−2^)	$\alpha_{0}$
5.182	0.244	186.4	0.344	6.75	0.291	0.342

## Data Availability

The data presented in this study are available on request from the corresponding author. The data are not publicly available due to the data also form part of an ongoing study.
